# Association of genetic variants with dyslipidemia and chronic kidney disease in a longitudinal population-based genetic epidemiological study

**DOI:** 10.3892/ijmm.2015.2152

**Published:** 2015-03-20

**Authors:** YOSHIJI YAMADA, KOTA MATSUI, ICHIRO TAKEUCHI, TETSUO FUJIMAKI

**Affiliations:** 1 Department of Human Functional Genomics, Life Science Research Center, Mie University, Tsu, Mie 514-8507, Japan; 2 Core Research for Evolutionary Science and Technology (CREST), Japan Science and Technology Agency, Tokyo 102-0076, Japan; 3 Department of Scientific and Engineering Simulation, Graduate School of Engineering, Nagoya Institute of Technology, Nagoya 466-8555, Japan; 4 Department of Cardiovascular Medicine, Inabe General Hospital, Inabe, Mie 511-0428, Japan

**Keywords:** dyslipidemia, chronic kidney disease, genetics, polymorphism, longitudinal study

## Abstract

We previously identified 9 genes and chromosomal region 3q28 as susceptibility loci for myocardial infarction, ischemic stroke, or chronic kidney disease (CKD) in Japanese individuals by genome-wide or candidate gene association studies. In the present study, we examined the association of 13 polymorphisms at these 10 loci with the prevalence of hypertriglyceridemia, hyper-low-density lipoprotein (LDL) cholesterolemia, hypo-high-density lipoprotein (HDL) cholesterolemia, or CKD in community-dwelling Japanese individuals. The study subjects comprised 6,027 individuals who were recruited to the Inabe Health and Longevity Study, a longitudinal genetic epidemiological study of atherosclerotic, cardiovascular and metabolic diseases. The subjects were recruited from individuals who visited the Health Care Center at Inabe General Hospital for an annual health checkup, and they were followed up each year (mean follow-up period, 5 years). Longitudinal analysis with a generalized estimating equation and with adjustment for covariates revealed that rs6929846 of butyrophilin, subfamily 2, member A1 gene (*BTN2A1*) was significantly associated with the prevalence of hypertriglyceridemia (P=0.0001), hyper-LDL cholesterolemia (P=0.0004), and CKD (P=0.0007); rs2569512 of interleukin enhancer binding factor 3 (*ILF3*) was associated with hyper-LDL cholesterolemia (P=0.0029); and rs2074379 (P=0.0019) and rs2074388 (P=0.0029) of alpha-kinase 1 (*ALPK1)* were associated with CKD. Longitudinal analysis with a generalized linear mixed-effect model and with adjustment for covariates among all individuals revealed that rs6929846 of *BTN2A1* was significantly associated with the serum concentrations of triglycerides (P=0.0011), LDL cholesterol (P=3.3×10^−5^), and creatinine (P=0.0006), as well as with the estimated glomerular filtration rate (eGFR) (P=0.0004); rs2569512 of *ILF3* was shown to be associated with the serum concentration of LDL cholesterol (P=0.0221); and rs2074379 (P=0.0302) and rs2074388 (P=0.0336) of *ALPK1* were shown to be associated with the serum concentration of creatinine. Similar analysis among individuals not taking any anti-dyslipidemic medication revealed that rs6929846 of *BTN2A1* was significantly associated with the serum concentrations of triglycerides (P=8.3×10^−5^) and LDL cholesterol (P=0.0004), and that rs2569512 of *ILF3* was associated with the serum concentration of LDL cholesterol (P=0.0010). *BTN2A1* may thus be a susceptibility gene for hypertriglyceridemia, hyper-LDL cholesterolemia and CKD in Japanese individuals.

## Introduction

Dyslipidemia, including hypertriglyceridemia, hyper-low-density lipoprotein (LDL) cholesterolemia and hypo-high-density lipoprotein (HDL) cholesterolemia, is a multifactorial disorder that results from an interaction between an individual’s genetic background, as well as lifestyle and environmental factors, the latter including a high-fat and high-calorie diet and physical inactivity (1,2). Given that dyslipidemia is an important risk factor for coronary artery disease, ischemic stroke and chronic kidney disease (CKD) (3,4), the personalized prevention of dyslipidemia is a key public health goal.

Recent genome-wide association studies (GWAS) have implicated various genes and loci in the predisposition to dyslipidemia in Caucasian populations (5–9). Although we have previously demonstrated that the apolipoprotein A–V gene is a susceptibility locus for dyslipidemia in Japanese individuals (10–12), the genes that contribute to genetic susceptibility to this condition in the Japanese population remain to be identified definitively.

CKD is a global public health concern, with affected individuals being at an increased risk not only for end-stage renal disease (ESRD), but also for a poor cardiovascular outcome and premature death (13–15). Disease prevention is an important strategy for reducing the overall burden of CKD and ESRD, with the identification of markers for disease risk being key both for risk prediction and for potential intervention to reduce the chance of future cardiovascular events (16).

In addition to conventional risk factors, such as diabetes mellitus and hypertension, prevoius studies have demonstrated the importance of genetic factors and of interactions between multiple genes and environmental factors in the development of CKD (17,18). Although recent GWAS have implicated various genes and loci in renal function or predisposition to CKD or ESRD in Caucasian (19–23) or African-American (24,25) populations, or in renal function-related traits in East Asian populations (26), the genes that contribute to genetic susceptibility to CKD in Japanese individuals remain to be identified definitively.

We previously identified 9 genes and the chromosomal region 3q28 as susceptibility loci for myocardial infarction, ischemic stroke, or CKD in Japanese individuals by genome-wide (27–29) or candidate gene (30–32) association studies. In the present study, we examined the possible association of 13 single nucleotide polymorphisms (SNPs) at these 10 loci with the prevalence of dyslipidemia (hypertriglyceridemia, hyper-LDL cholesterolemia, or hypo-HDL cholesterolemia) or CKD in community-dwelling Japanese individuals.

## Materials and methods

### Study population

The study subjects comprised a total of 6,027 community-dwelling individuals who were recruited to a population-based cohort study (Inabe Health and Longevity Study) in Inabe (Mie, Japan). The Inabe Health and Longevity Study is a longitudinal genetic epidemiological study of atherosclerotic, cardiovascular, and metabolic diseases (33–39). The detailed methods for the recruitment of the study subjects and the collection and storage of medical examination data and genomic DNA samples have been described in a previous study of ours (33).

For the dyslipidemia analysis, 3,790 subjects with dyslipidemia and 2,237 controls were examined. Venous blood was collected in the early morning after the subjects had fasted overnight. The blood samples were centrifuged at 1,600 × g for 15 min at 4°C, and serum was separated and stored at −30°C until analysis. The serum concentrations of triglycerides, LDL cholesterol and HDL cholesterol were measured using a standard method at a clinical laboratory in the hospital.

The subjects with dyslipidemia had either hypertriglyceridemia, hyper-LDL cholesterolemia, or hypo-HDL cholesterolemia. Individuals with hypertriglyceridemia and the corresponding controls had serum concentrations of triglycerides of ≥150 mg/dl (1.65 mmol/l) and of <150 mg/dl, respectively; individuals with hyper-LDL cholesterolemia and the corresponding controls had serum concentrations of LDL cholesterol of ≥140 mg/dl (3.64 mmol/l) and of <140 mg/dl, respectively; and individuals with hypo-HDL cholesterolemia and the corresponding controls had serum concentrations of HDL cholesterol of <40 mg/dl (1.04 mmol/l) and of ≥40 mg/dl, respectively. The control individuals had no history of dyslipidemia or of taking any anti-dyslipidemic medication.

For the CKD analysis, a total of 655 subjects with CKD and 1,457 controls were examined. The estimated glomerular filtration rate (eGFR) was calculated with the use of the simplified prediction equation derived from the modified version of that described in the Modification of Diet in Renal Disease (MDRD) Study, as proposed by the Japanese Society of Nephrology (40): eGFR (ml/min/1.73 m^2^) = 194 × [age (years)]^−0.287^ × [serum creatinine (mg/dl)]^−1.094^ × [0.739 if female]. The National Kidney Foundation Kidney Disease Outcomes Quality Initiative guidelines recommend a diagnosis of CKD if the eGFR was <60 ml/min/1.73 m^2^ (16). On the basis of this criterion, 655 subjects were diagnosed with CKD. The control subjects comprised 1,457 individuals whose eGFR was ≥90 ml/min/1.73 m^2^. The control individuals did not have functional or structural abnormalities of the kidneys or a history of renal disease. Although some control individuals had hypertension, diabetes mellitus, or dyslipidemia, they had no renal complications.

The study protocol complied with the Declaration of Helsinki and was approved by the Committees on the Ethics of Human Research of Mie University Graduate School of Medicine and Inabe General Hospital (Mie, Japan). Written informed consent was obtained from all subjects.

### Selection and genotyping of polymorphisms

The 13 SNPs examined in the present study were selected from our previous genome-wide (27–29) or candidate gene (30–32) association studies and were described previously (33). Wild-type (ancestral) and variant alleles of the SNPs were determined from the SNP database (dbSNP; National Center for Biotechnology Information, Bethesda, MD, USA).

Venous blood (5 ml) was collected into tubes containing 50 mmol/l ethylenediaminetetraacetic acid (disodium salt), and peripheral blood leukocytes were isolated and genomic DNA was extracted from these cells using a DNA extraction kit (SMITEST EX-R&D; Medical & Biological Laboratories, Co., Ltd., Aichi, Japan). Genotypes of the 13 SNPs were determined at G&G Science Co., Ltd. (Fukushima, Japan) by a method that combines the polymerase chain reaction and sequence-specific oligonucleotide probes with suspension array technology (Luminex Corp., Austin, TX, USA). Primers, probes, and other conditions for genotyping of the SNPs examined in the present study were as previously described (33), as was the detailed genotyping methodology (41).

### Statistical analysis

Quantitative data were compared between the subjects with dyslipidemia or CKD and the corresponding controls with the unpaired Student’s t-test. Categorical data were compared using the χ^2^ test. We examined the association of the 13 SNPs with dyslipidemia or CKD in a 5-year longitudinal cohort study. Longitudinal changes in the prevalence of hypertriglyceridemia, hyper-LDL cholesterolemia, hypo-HDL cholesterolemia, or CKD were compared between 2 groups (the dominant or recessive genetic model) by a generalized estimating equation (42) and with adjustment for age, gender and body mass index (BMI) for the analysis of dyslipidemia, or for age, gender, BMI, smoking status, and the prevalence of hypertension, diabetes mellitus and dyslipidemia for the CKD analysis. Longitudinal changes in the serum concentrations of triglycerides, LDL cholesterol, HDL cholesterol, or creatinine or in the eGFR in all individuals (or in individuals not taking any anti-dyslipidemic medication for the dyslipidemia analysis) were compared between 2 groups (the dominant or recessive model) in a generalized linear mixed-effect model (43) with adjustment for the same corresponding covariates. Age-related changes in the prevalence of hypertriglyceridemia, hyper-LDL cholesterolemia, or CKD; in the serum concentrations of triglycerides, LDL cholesterol, or creatinine; or in the eGFR were estimated with quadratic curve controlling for the observation year. A value of P<0.05 was considered to indicate a statistically significant difference. Statistical analysis was performed using R Software version 3.0.2 (the R Project for Statistical Computing) and JMP Genomics version 6.0 (SAS Institute, Inc., Cary, NC, USA).

## Results

### Analysis of dyslipidemia

All the characteristics of the 3,790 subjects with dyslipidemia and the 2,237 controls in the cross-sectional analysis in March 2014 are shown in Table I. Age, the frequency of the male gender and BMI were significantly greater in the subjects with dyslipidemia than in the controls.

The association of the 13 SNPs with the prevalence of hypertriglyceridemia, hyper-LDL cholesterolemia, or hypo-HDL cholesterolemia was analyzed with a generalized estimating equation and with adjustment for age, gender and BMI (Table II). The rs6929846 (T→C) SNP of the butyrophilin, subfamily 2, member A1 gene (*BTN2A1*) was found to be significantly associated with the prevalence of both hypertriglyceridemia (dominant model) and hyper-LDL cholesterolemia (dominant and recessive models). The rs2569512 (G→A) SNP of the interleukin enhancer binding factor 3, 90 kDa gene (*ILF3*) was also significantly associated with hyper-LDL cholesterolemia (recessive model). Genotype distributions for rs6929846 and rs2569512 in the subjects with hypertriglyceridemia or hyper-LDL cholesterolemia and the corresponding controls for 5-year longitudinal data are shown in Table III.

Given that rs6929846 and rs2569512 were found to be significantly associated with hypertriglyceridemia or hyper-LDL cholesterolemia, the association of these SNPs with the serum concentrations of triglycerides or LDL cholesterol in all the individuals or in the individuals not taking any anti-dyslipidemic medication was analyzed with a generalized linear mixed-effect model and with adjustment for age, gender and BMI (Table IV). The rs6929846 SNP of *BTN2A1* was significantly associated with the serum concentrations of both triglycerides (dominant model) and LDL cholesterol (dominant and recessive models) among all the individuals and in the individuals not taking any anti-dyslipidemic medication, with the minor *T* allele being associated with increased serum triglyceride and LDL cholesterol levels. The rs2569512 SNP of *ILF3* was also associated with the serum concentrations of LDL cholesterol among all the individuals (dominant and recessive models) and in the individuals not taking any anti-dyslipidemic medication (recessive model), with the minor *A* allele being associated with lower concentrations of serum LDL cholesterol.

**Figure 1:**
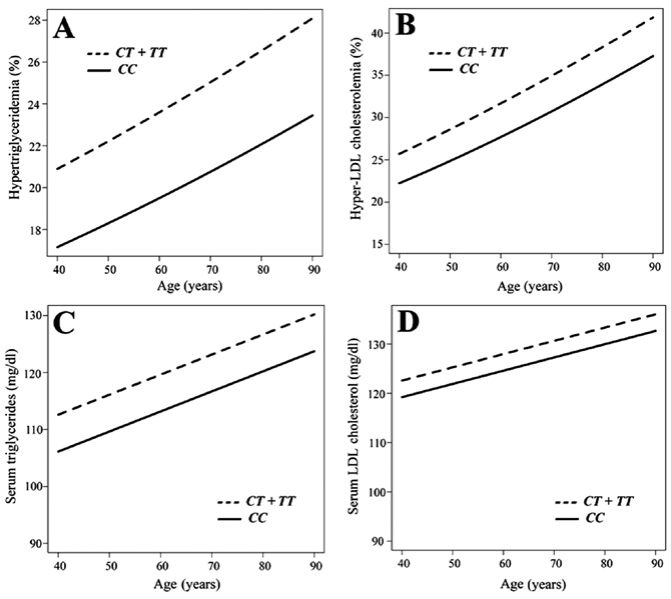
Longitudinal analysis of the association between the prevalence of (A) hypertriglyceridemia or (B) hyper-low-density lipoprotein (LDL) cholesterolemia and age with a generalized estimating equation, or between the serum concentrations of (C) triglycerides or (D) LDL cholesterol and age with a generalized linear mixed-effect model, according to the genotype for rs6929846 of butyrophilin, subfamily 2, member A1 gene (*BTN2A1*) (*CT* + *TT* vs. *CC*).

The association between the prevalence of hypertriglyceridemia or hyper-LDL cholesterolemia and age was analyzed longitudinally with a generalized estimating equation according to the *BTN2A1* genotype. The prevalence of hypertriglyceridemia (Fig. 1A) or hyper-LDL cholesterolemia (Fig. 1B) was greater in the combined group of subjects with the *CT* or *TT* genotypes of rs6929846 of *BTN2A1* than in the subjects with the *CC* genotype from 40 to 90 years of age. The association between the serum concentrations of triglycerides or LDL cholesterol with age was also analyzed longitudinally according to the *BTN2A1* genotype in all individuals with a generalized linear mixed-effect model. The serum concentrations of triglycerides (Fig. 1C) or LDL cholesterol (Fig. 1D) were greater in the combined group of individuals with the *CT* or *TT* genotypes of rs6929846 of *BTN2A1* than in those with the *CC* genotype from 40 to 90 years of age.

### CKD analysis

The characteristics of the 655 subjects with CKD and the 1,457 controls in the cross-sectional analysis carried out in March 2014 are shown in Table V. Age, the frequency of the male gender and BMI were significantly greater in the subjects with CKD than in the controls.

The association of the 13 SNPs with the prevalence of CKD was analyzed with a generalized estimating equation and with adjustment for age, gender, BMI, smoking status, and the prevalence of hypertension, diabetes mellitus and dyslipidemia (Table VI). The rs2074379 (G→A) and rs2074388 (G→A) SNPs of the α-kinase 1 gene (*ALPK1*) (dominant model) as well as rs6929846 (T→C) of *BTN2A1* (dominant model) were found to be significantly associated with the prevalence of CKD. The genotype distributions for rs2074379, rs2074388 and rs6929846 in the subjects with CKD and the controls for 5-year longitudinal data are shown in Table VII.

Given that 3 SNPs were found to be significantly associated with CKD, the association of these SNPs with the serum concentration of creatinine or with the eGFR in all the individuals (n=6,027) was analyzed with a generalized linear mixed-effect model and with adjustment for age, gender, BMI, smoking status, and the prevalence of hypertension, diabetes mellitus and dyslipidemia (Table VIII). The rs2074379 and rs2074388 SNPs of *ALPK1* were significantly associated with the serum concentration of creatinine (dominant model), with the minor *G* allele of each SNP being associated with increased serum creatinine levels. The rs6929846 SNP of *BTN2A1* (dominant model) was significantly associated with the serum concentration of creatinine and the eGFR, with the minor *T* allele being associated with an increased creatinine level and a lower eGFR.

**Figure 2:**
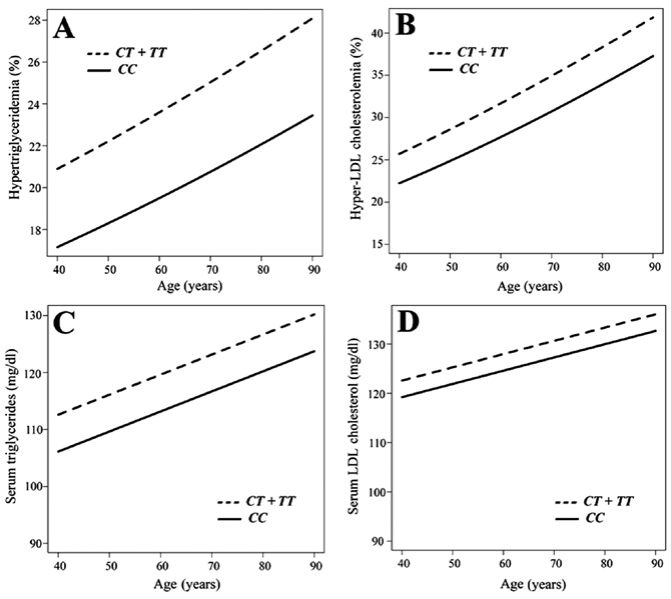
Longitudinal analysis of the association between the prevalence of chronic kidney disease (CKD) and age with (A) a generalized estimating equation, or (B) between the serum concentration of creatinine or (C) estimated glomerular filtration rate (eGFR) and age with a generalized linear mixed-effect model, according to the genotype for rs6929846 of butyrophilin, subfamily 2, member A1 gene (*BTN2A1*) (*CT* + *TT* vs. *CC*).

The association between the prevalence of CKD and age was analyzed longitudinally with a generalized estimating equation according to the *BTN2A1* genotype. The prevalence of CKD was greater in the combined group of subjects with the *CT* or *TT* genotypes of rs6929846 of *BTN2A1* than in the subjects with the *CC* genotype from 40 to 90 years of age (Fig. 2A). The association between the serum concentration of creatinine or the eGFR with age was also analyzed longitudinally according to the *BTN2A1* genotype in all individuals with a generalized linear mixed-effect model. The serum concentration of creatinine was greater (Fig. 2B), whereas the eGFR was lower (Fig. 2C), in the combined group of individuals with the *CT* or *TT* genotypes of rs6929846 of *BTN2A1* than in those with the *CC* genotype from 40 to 90 years of age.

## Discussion

Given that genetic factors, as well as interactions between multiple genes and environmental factors have been previously shown to be important in the development of dyslipidemia and CKD (1,2,17,18), the prediction of the risk for these conditions on the basis of genetic variants would be beneficial for the personalized prevention of these conditions. In the present study, we demonstrated that rs6929846 of *BTN2A1* was significantly associated with the prevalence of hypertriglyceridemia, hyper-LDL cholesterolemia and CKD in a longitudinal genetic epidemiological study, with the minor *T* allele of this SNP representing a risk factor for these conditions. In previous studies of ours, we demonstrated that rs6929846 of *BTN2A1* was significantly associated with dyslipidemia (44) and CKD (45) in cross-sectional studies of different hospital-based populations. We have also previously detected an association of this SNP with dyslipidemia (38) and CKD (36) in previous cross-sectional analyses of the Inabe Health and Longevity Study. The results of the present longitudinal population-based study are thus consistent with these previous observations (36,38,44,45) and validate the association of rs6929846 of *BTN2A1* with dyslipidemia and CKD.

BTN2A1 is a cell-surface transmembrane glycoprotein and a member of the butyrophilin superfamily of proteins. Many of these proteins regulate immune function, and polymorphisms within the coding sequences of the corresponding genes have been associated with the predisposition to inflammatory diseases (46). We have previously demonstrated that the *T* allele of rs6929846 of *BTN2A1* is associated with an increased risk of myocardial infarction and with an increased transcriptional activity of *BTN2A1* (27). We have also previously demonstrated that the serum concentration of high-sensitivity C-reactive protein is significantly greater in individuals in the combined group of *CT* or *TT* genotypes for this SNP than in those with the *CC* genotype among healthy subjects (47). These observations suggest that the *T* allele of rs6929846 of *BTN2A1* may accelerate inflammatory processes.

Inflammation has been found to induce multiple alterations in lipid and lipoprotein metabolism. Chronic inflammatory conditions thus result in increased serum concentrations of triglycerides and LDL cholesterol and in lower serum HDL cholesterol levels (48,49). Multiple cytokines likely affect the metabolism of cholesterol or triglycerides by several mechanisms, including the increased production and the reduced clearance of very low density lipoproteins, impaired reverse cholesterol transport and the reduced excretion of bile acids (48,50,51). The acceleration of inflammatory processes by the *T* allele of rs6929846 may thus result in changes in lipid metabolism, leading to hypertriglyceridemia and hyper-LDL cholesterolemia, although the underlying mechanisms remain to be elucidated.

Renal tubulointerstitial fibrosis associated with injured tubules and inflammatory leukocytes has been considered a common characteristic of CKD (52). Chronic inflammation plays a fundamental role in the promotion of interlinked fibrosis and cellular injury within the tubulointerstitium, with macrophages initially mediating this inflammatory process (53). In addition, macrophage infiltration in response to glomerular and tubular injury leads to the production of pro-inflammatory cytokines, vasoactive eicosanoids and reactive oxygen species (54,55). This vicious cascade accelerates structural and functional damage, finally leading to the deterioration of renal function. Given the role of chronic inflammation in the pathogenesis of CKD, the association of rs6929846 of *BTN2A1* with CKD may be attributable to the acceleration of inflammatory processes by the *T* allele of this polymorphism.

The results from the present study demonstrate that the rs2569512 SNP of *ILF3* and both the rs2074379 and rs2074388 SNPs of *ALPK1* are associated with hyper-LDL cholesterolemia and CKD, respectively. *ILF3* has previously been found to be a candidate gene for myocardial infarction in Japanese individuals (27). ILF3 is a subunit of nuclear factor of activated T cells (NFAT), a transcription factor required for the expression of the interleukin-2 gene in T cells (56). ILF3 plays a role in the regulation of transcription, translation, mRNA stability and primary microRNA processing (57). It is overexpressed in nasopharyngeal cancer, non-small cell lung cancer and ovarian cancer, suggesting that ILF3 may contribute to carcinogenesis (57). The functional relevance of rs2569512 of *ILF3* to the metabolism of LDL cholesterol, however, remains unclear. *ALPK1* was previously identified as a susceptibility gene for CKD among subjects with diabetes mellitus (29). ALPK1 is thought to act synergistically with monosodium urate monohydrate crystals to promote the production of pro-inflammatory cytokines through the activation of nuclear factor-κB and mitogen-activated protein kinase (ERK1/2 and p38) signaling in cultured HEK293 cells, indicating that ALPK1 may contribute to inflammation associated with the development of gout (58). ALPK1 may thus promote chronic inflammation of the kidneys, although the functional relevance of SNPs of *ALPK1* to the pathogenesis of CKD remains unclear.

There were some limitations to the present study: ⅰ) given that the results of the present study were not replicated, validation of our findings will require their replication with other independent subject panels or ethnic groups; ⅱ) it is possible that rs6929846 of *BTN2A1* is in linkage disequilibrium with other polymorphisms in the same gene or in nearby genes that are actually responsible for the development of dyslipidemia or CKD; and ⅲ) the functional relevance of rs6929846 of *BTN2A1* to the pathogenesis of dyslipidemia or CKD has not been determined.

In conclusion, the results from the present study suggest that *BTN2A1* is a susceptibility gene for hypertriglyceridemia, hyper-LDL cholesterolemia and CKD in community-dwelling Japanese individuals. The determination of genotypes for rs6929846 of *BTN2A1* may prove informative for the assessment of the genetic risk for these conditions in the Japanese population.

## Figures and Tables

**Table I:** Characteristics of the subjects with dyslipidemia and the controls: cross-sectional analysis in March 2014.

Parameter	Dyslipidemia^a^	Controls^a^	P-value
No. of subjects	3,790	2,237	
Age (years)	56.8±11.8 (3,790)	49.8±13.6 (2,237)	<0.0001
Gender (male/female, %)	60.5/39.5 (3,790)	47.3/52.7 (2,237)	<0.0001
Height (cm)	162.5±9.5 (3,752)	162.5±8.7 (2,202)	0.8411
Weight (kg)	62.9±12.6 (3,751)	57.6±10.6 (2,201)	<0.0001
BMI (kg/m^2^)	23.7±3.4 (3,751)	21.7±3.0 (2,201)	<0.0001
Waist circumference (cm)	82.6±9.0 (3,524)	76.8±8.5 (2,081)	<0.0001
Alcohol consumption (%)	46.9 (3,790)	50.5 (2,237)	0.0073
Current or former smoker (%)	49.6 (3,790)	39.1 (2,237)	<0.0001
Systolic blood pressure (mmHg)	123±16 (3,746)	116±16 (2,199)	<0.0001
Diastolic blood pressure (mmHg)	76±12 (3,746)	72±12 (2,199)	<0.0001
Mean blood pressure (mmHg)	92±12 (3,746)	86±12 (2,199)	<0.0001
Ocular tension (right, mmHg)	13.7±3.0 (1,323)	13.2±2.9 (738)	0.0015
Functional vital capacity (l)	3.30±0.83 (1,435)	3.30±0.78 (808)	0.9768
FEV1% (%)	80.7±6.3 (1,435)	82.3±6.8 (808)	<0.0001
Serum albumin (g/l)	44.9±2.7 (2,761)	44.1±2.6 (1,451)	<0.0001
Serum total cholesterol (mg/dl)	211±35 (3,772)	185±25 (2,178)	<0.0001
Serum triglycerides (mg/dl)	135±86 (3,761)	72±26 (2,175)	<0.0001
Serum LDL cholesterol (mg/dl)	133±32 (3,758)	105±20 (2,174)	<0.0001
Serum HDL cholesterol (mg/dl)	59.9±16.2 (3,759)	71.4±16.9 (2,175)	<0.0001
Fasting plasma glucose (mmol/l)	5.73±1.28 (3,775)	5.34±0.84 (2,181)	<0.0001
Blood hemoglobin A_1c_ (%)	5.79±0.75 (2,892)	5.51±0.50 (1,571)	<0.0001
Blood urea nitrogen (mmol/l)	5.28±1.98 (2,686)	5.08±2.05 (1,415)	0.0024
Serum creatinine (mg/dl)	0.86±0.97 (3,592)	0.79±0.81 (1,984)	0.0046
eGFR (ml/min/1.73 m^2^)	74.8±16.4 (3,592)	80.0±16.9 (1,984)	<0.0001
Serum uric acid (*μ*mol/l)	339±85 (3,572)	303±82 (1,959)	<0.0001
Serum C-reactive protein (*μ*g/l)	1327±7465 (1,354)	959±4049 (759)	0.2084
White blood cells (10^3^ cells/*μ*l)	5.58±1.75 (2,799)	5.06±1.48 (1,808)	<0.0001
Red blood cells (10^4^ cells/*μ*l)	443±45 (2,807)	427±42 (1,816)	<0.0001
Hemoglobin (g/l)	140±15 (2,807)	134±15 (1,816)	<0.0001
Hematocrit (%)	40.8±4.2 (2,805)	39.3±4.1 (1,813)	<0.0001
Platelets (10^4^ cells/*μ*l)	22.5±5.4 (2,780)	21.9±5.4 (1,788)	0.0002

Quantitative data are the means ± SD.

a Values in parentheses indicate the numbers of measurements taken. BMI, body mass index; FEV1%, forced expiratory volume in 1 sec percentage; LDL, low-density lipoprotein; HDL, high-density lipoprotein; eGFR, estimated glomerular filtration rate.

**Table II:** Association of the 13 polymorphisms with hypertriglyceridemia, hyper-LDL cholesterolemia, or hypo-HDL cholesterolemia analyzed for 5-year longitudinal data with a generalized estimating equation.

Gene or locus	SNP	Hypertriglyceridemia		Hyper-LDL cholesterolemia		Hypo-HDL cholesterolemia	
		P-value^a^	P-value^b^	P-value^a^	P-value^b^	P-value^a^	P-value^b^
*FAM78B*	rs2116519 (C→T)	0.4656	0.7725	0.0808	0.0658	0.7946	0.8187
3q28	rs9846911 (A→G)	0.7176	0.1923	0.9092	0.8869	0.2810	0.6875
*ALPK1*	rs2074379 (G→A)	0.9341	0.5329	0.4143	0.1189	0.3001	0.8486
*ALPK1*	rs2074380 (G→A)	0.9752	0.6585	0.0707	0.1150	0.4270	0.7847
*ALPK1*	rs2074381 (A→G)	0.8979	0.3325	0.0629	0.2752	0.3514	0.7445
*ALPK1*	rs2074388 (G→A)	0.8611	0.4981	0.4067	0.1012	0.3006	0.8762
*BTN2A1*	rs6929846 (T→C)	**0.0001**	0.9248	**0.0004**	**0.0092**	0.0690	0.0582
*THBS2*	rs8089 (T→G)	0.5220	0.8496	0.3422	0.9957	0.5382	0.3849
*PDX1*	rs146021107 (G→-)	0.9664	0.8917	0.6197	0.7836	0.9954	0.8950
*F7*	rs6046 (G→A)	0.4703	0.2638	0.1146	0.2668	0.1596	0.1130
*LLGL2*	rs1671021 (G→A)	0.4416	0.8355	0.1622	0.6364	0.3936	0.3351
*ILF3*	rs2569512 (G→A)	0.0510	0.4616	0.5391	**0.0029**	0.5438	0.9550
*CELSR1*	rs6007897 (C→T)	0.8546	ND	0.8124	ND	0.5352	ND

The prevalence of hypertriglyceridemia, hyper-LDL cholesterolemia, or hypo-HDL cholesterolemia was compared between 2 groups (dominant or recessive model) for each polymorphism with adjustment for age, gender and BMI. P-values of <0.05 are shown in bold.

a Dominant: *AA* vs. *AB* + *BB* (*A*, major allele; *B*, minor allele).

b Recessive: *AA* + *AB* vs. *BB*. LDL, low-density lipoprotein; HDL, high-density lipoprotein; BMI, body mass index; SNP, single nucleotide polymorphism; *ALPK1*, α-kinase 1 gene; *BTN2A1*, butyrophilin, subfamily 2, member A1 gene; *THBS2*, thrombospondin 2 gene; *PDX1*, pancreatic and duodenal homeobox 1; *F7*, coagulation factor VII (serum prothrombin conversion accelerator); *LLGL2*, lethal giant larvae homolog 2 (*Drosophila*); *ILF3*, interleukin enhancer binding factor 3, 90 kDa; *CELSR1*, cadherin, EGF LAG seven-pass G-type receptor 1; ND, not determined.

**Table III:** Genotype distributions for rs6929846 of *BTN2A1* and rs2569512 of *ILF3* among individuals with hypertriglyceridemia or hyper-LDL cholesterolemia, as well as the corresponding controls analyzed for 5-year longitudinal data with a generalized estimating equation.

Gene	SNP	Genotype	Hypertriglyceridemia^a^	Controls^a^	Hyper-LDL cholesterolemia^a^	Controls^a^
*BTN2A1*	rs6929846 (T→C)					
		*CC*	3,998 (74.8)	17,595 (79.3)	5,632 (76.0)	15,068 (79.5)
		*CT*	1,281 (24.0)	4,296 (19.4)	1,643 (22.2)	3,677 (19.4)
		*TT*	66 (1.2)	284 (1.3)	133 (1.8)	212 (1.1)
*ILF3*	rs2569512 (G→A)					
		*GG*	2,423 (45.3)	9,566 (43.1)	3,264 (44.1)	8,257 (43.6)
		*GA*	2,385 (44.6)	10,249 (46.2)	3,477 (46.9)	8,591 (45.3)
		*AA*	537 (10.0)	2,360 (10.6)	667 (9.0)	2,109 (11.1)

a Values indicate the numbers of measurements taken, with the percentages shown in parentheses. *BTN2A1*, butyrophilin, subfamily 2, member A1 gene; *ILF3*, interleukin enhancer binding factor 3, 90 kDa; LDL, low-density lipoprotein; SNP, single nucleotide polymorphism.

**Table IV:** Association of the polymorphisms with serum concentrations of triglycerides or LDL cholesterol in all individuals or in individuals not taking any anti-dyslipidemic medication analyzed for 5-year longitudinal data with a general linear mixed-effect model.

Gene	SNP	Parameter (mg/dl)	Dominant model^a^		P-value	Recessive model^a^		P-value
All individuals								
* BTN2A1*	rs6929846 (T→C)		*CC* (21,595)	*CT* + *TT* (5,927)		*CC* + *CT* (27,170)	*TT* (350)	
		Serum triglycerides	110.1±80.7	117.1±78.9	**0.0011**	111.6±80.5	113.7±68.1	0.1995
* BTN2A1*	rs6929846 (T→C)		*CC* (20,700)	*CT* + *TT* (5,665)		*CC* + *CT* (26,020)	*TT* (345)	
		Serum LDL cholesterol	122.8±30.6	126.4±31.1	**3.3×10^−5^**	123.4±30.7	133.3±32.2	**0.0015**
* ILF3*	rs2569512 (G→A)		*GG* (11,521)	*GA* + *AA* (14,844)		*GG* + *GA* (23,589)	*AA* (2,776)	
		Serum LDL cholesterol	124.4±30.5	122.8±30.9	**0.0332**	123.9±30.8	120.8±29.6	**0.0221**
Individuals not taking any anti-dyslipidemic medication								
* BTN2A1*	rs6929846 (T→C)		*CC* (20,850)	*CT* + *TT* (5,691)		*CC* + *CT* (26,211)	*TT* (330)	
		Serum triglycerides	109.4±80.7	116.7±79.3	**8.3×10^−5^**	110.9±80.6	113.6±69.1	0.8613
* BTN2A1*	rs6929846 (T→C)		*CC* (19,957)	*CT* + *TT* (5,429)		*CC* + *CT* (25,061)	*TT* (325)	
		Serum LDL cholesterol	122.9±30.6	126.6±31.0	**0.0004**	123.6±30.7	134.1±32.4	**0.0159**
* ILF3*	rs2569512 (G→A)		*GG* (11,089)	*GA* + *AA* (14,297)		*GG* + *GA* (22,717)	*AA* (2,669)	
		Serum LDL cholesterol	124.6±30.6	123.0±30.9	0.4235	124.1±30.9	121.0±29.6	**0.0010**

Serum concentrations of triglycerides or LDL cholesterol were compared between 2 groups (dominant or recessive model) for each polymorphism with adjustment for age, gender and BMI. Data for serum concentrations of triglycerides or LDL cholesterol are the means ± SD. P-values of <0.05 are shown in bold.

a Values in parentheses indicate the numbers of measurements taken. LDL, low-density lipoprotein; BMI, body mass index; SNP, single nucleotide polymorphism; *BTN2A1*, butyrophilin, subfamily 2, member A1 gene; *ILF3*, interleukin enhancer binding factor 3, 90 kDa.

**Table V:** Characteristics of the subjects with chronic kidney disease and controls: cross-sectional analysis in March 2014.

Parameter	CKD^a^	Controls^a^	P-value
No. of subjects	655	1,457	
Age (years)	66.7±9.5 (655)	46.3±12.9 (1,457)	<0.0001
Gender (male/female, %)	64.6/35.4 (655)	50.2/49.8 (1,457)	<0.0001
Height (cm)	160.5±9.3 (620)	162.9±9.0 (1,425)	<0.0001
Weight (kg)	60.8±11.6 (618)	60.4±12.6 (1,425)	0.4654
BMI (kg/m^2^)	23.5±3.2 (618)	22.7±3.7 (1,425)	<0.0001
Waist circumference (cm)	82.6±9.1 (499)	79.3±9.8 (1,371)	<0.0001
Alcohol consumption (%)	41.5 (655)	47.7 (1,457)	0.0084
Current or former smoker (%)	43.7 (655)	42.4 (1,457)	0.5918
Systolic blood pressure (mmHg)	126±17 (612)	117±16 (1,425)	<0.0001
Diastolic blood pressure (mmHg)	76±12 (612)	72±12 (1,425)	<0.0001
Mean blood pressure (mmHg)	93±12 (612)	87±13 (1,425)	<0.0001
Ocular tension (right, mmHg)	13.3±3.1 (173)	13.9±2.9 (382)	0.0162
Functional vital capacity (l)	3.04±0.74 (181)	3.29±0.76 (415)	0.0002
FEV1% (%)	79.0±6.9 (181)	82.5±6.8 (415)	<0.0001
Serum albumin (g/l)	43.6±3.7 (570)	44.8±2.6 (707)	<0.0001
Serum total cholesterol (mg/dl)	196±38 (640)	198±35 (1,399)	0.1229
Serum triglycerides (mg/dl)	117±60 (625)	105±88 (1,400)	0.0016
Serum LDL cholesterol (mg/dl)	119±31 (622)	118±32 (1,399)	0.3852
Serum HDL cholesterol (mg/dl)	59.8±17.4 (623)	65.6±17.3 (1,400)	<0.0001
Fasting plasma glucose (mmol/l)	5.89±1.42 (647)	5.58±1.39 (1,399)	<0.0001
Blood hemoglobin A_1c_ (%)	5.86±0.65 (561)	5.65±0.86 (875)	<0.0001
Blood urea nitrogen (mmol/l)	7.25±3.86 (564)	4.47±1.18 (670)	<0.0001
Serum creatinine (mg/dl)	1.69±2.49 (655)	0.60±0.10 (1,006)	<0.0001
eGFR (ml/min/1.73 m^2^)	48.9±14.5 (655)	100.5±9.9 (1,006)	<0.0001
Serum uric acid (*μ*mol/l)	376±92 (647)	297±77 (1,003)	<0.0001
Serum C-reactive protein (*μ*g/l)	3224±16767 (228)	801±2049 (381)	0.0055
White blood cells (10^3^ cells/*μ*l)	5.43±2.18 (424)	5.52±1.67 (1,172)	0.3943
Red blood cells (10^4^ cells/*μ*l)	414±53 (426)	440±42 (1,179)	<0.0001
Hemoglobin (g/l)	132±18 (426)	137±16 (1,179)	<0.0001
Hematocrit (%)	38.5±5.0 (426)	40.1±4.4 (1,174)	<0.0001
Platelets (10^4^ cells/*μ*l)	20.1±5.6 (420)	23.2±5.6 (1,163)	<0.0001

Quantitative data are the means ± SD.

a Values in parentheses indicate the numbers of measurements taken. CKD, chronic kidney disease; BMI, body mass index; FEV1%, forced expiratory volume in 1 sec percentage; LDL, low-density lipoprotein; HDL, high-density lipoprotein; eGFR, estimated glomerular filtration rate.

**Table VI:** Association of 13 polymorphisms with CKD analyzed for 5-year longitudinal data with a generalized estimating equation.

Gene or locus	SNP	P-value^a^	P-value^b^
*FAM78B*	rs2116519 (C→T)	0.9541	0.3357
3q28	rs9846911 (A→G)	0.6325	0.1752
*ALPK1*	rs2074379 (G→A)	**0.0019**	0.1824
*ALPK1*	rs2074380 (G→A)	0.0610	0.4038
*ALPK1*	rs2074381 (A→G)	0.1032	0.2770
*ALPK1*	rs2074388 (G→A)	**0.0029**	0.1193
*BTN2A1*	rs6929846 (T→C)	**0.0007**	0.1230
*THBS2*	rs8089 (T→G)	0.3192	0.2006
*PDX1*	rs146021107 (G→-)	0.4138	0.1905
*F7*	rs6046 (G→A)	0.3869	0.9015
*LLGL2*	rs1671021 (G→A)	0.4093	0.5071
*ILF3*	rs2569512 (G→A)	0.5149	0.8341
*CELSR1*	rs6007897 (C→T)	0.3544	ND

The prevalence of CKD was compared between 2 groups (dominant or recessive model) for each polymorphism with adjustment for age, gender, BMI, smoking status, and the prevalence of hypertension, diabetes mellitus and dyslipidemia. P-values of <0.05 are shown in bold.

a Dominant: *AA* vs. *AB* + *BB* (*A*, major allele; *B*, minor allele).

b Recessive: *AA* + *AB* vs. *BB*. CKD, chronic kidney disease; BMI, body mass index; SNP, single nucleotide polymorphism; *ALPK1*, α-kinase 1 gene; *BTN2A1*, butyrophilin, subfamily 2, member A1 gene; *THBS2*, thrombospondin 2 gene; *PDX1*, pancreatic and duodenal homeobox 1; *F7*, coagulation factor VII (serum prothrombin conversion accelerator); *LLGL2*, lethal giant larvae homolog 2 (Drosophila); *ILF3*, interleukin enhancer binding factor 3, 90 kDa; *CELSR1*, cadherin, EGF LAG seven-pass G-type receptor 1 ND, not determined.

**Table VII:** Genotype distributions for rs2074379 and rs2074388 in *ALPK1* and for rs6929846 in *BTN2A1* among the subjects with CKD and the controls.

Gene	SNP	Genotype	CKD^a^	Controls^a^
*ALPK1*	rs2074379 (G→A)			
		*AA*	633 (42.0)	1,689 (45.6)
		*AG*	730 (48.4)	1,619 (43.7)
		*GG*	144 (9.6)	395 (10.7)
*ALPK1*	rs2074388 (G→A)			
		*AA*	634 (42.1)	1,690 (45.6)
		*AG*	723 (48.0)	1,611 (43.5)
		*GG*	150 (10.0)	402 (10.9)
*BTN2A1*	rs6929846 (T→C)			
		*CC*	1,136 (75.4)	2,925 (79.0)
		*CT*	341 (22.6)	721 (19.5)
		*TT*	30 (2.0)	57 (1.5)

a Values are the numbers of measurements taken, with the percentages shown in parentheses. *ALPK1*, α-kinase 1 gene; *BTN2A1*, butyrophilin, subfamily 2, member A1 gene; CKD, chronic kidney disease; SNP, single nucleotide polymorphism.

**Table VIII:** Association of 3 polymorphisms of *ALPK1* and *BTN2A1* with the serum concentration of creatinine or with the eGFR in all individuals analyzed for 5-year longitudinal data with a generalized linear mixed-effect model.

Gene	SNP	Parameter	Dominant model^a^		P-value	Recessive model^a^		P-value
*ALPK1*	rs2074379 (G→A)		*AA* (8,219)	*AG* + *GG* (9,569)		*AA* + *AG* (16,035)	*GG* (1,753)	
		Serum creatinine (mg/dl)	0.731±0.169	0.737±0.193	**0.0302**	0.734±0.183	0.731±0.175	0.9110
		eGFR (ml/min/1.73 m^2^)	79.0±14.7	78.9±15.7	0.3163	78.9±15.2	79.6±15.8	0.8386
*ALPK1*	rs2074388 (G→A)		*AA* (8,216)	*AG* + *GG* (9,572)		*AA* + *AG* (16,028)	*GG* (1,760)	
		Serum creatinine (mg/dl)	0.731±0.169	0.737±0.193	**0.0336**	0.734±0.183	0.732±0.175	0.9083
		eGFR (ml/min/1.73 m^2^)	79.0±14.7	78.9±15.7	0.3345	78.9±15.2	79.6±15.9	0.8178
*BTN2A1*	rs6929846 (T→C)		*CC* (13,862)	*CT* + *TT* (3,926)		*CC* + *CT* (17,519)	*TT* (269)	
		Serum creatinine (mg/dl)	0.732±0.173	0.742±0.212	**0.0006**	0.734±0.182	0.727±0.191	0.3911
		eGFR (ml/min/1.73 m^2^)	79.1±15.2	78.4±15.4	**0.0004**	78.9±15.2	79.8±18.7	0.6394

The serum concentration of creatinine or eGFR was compared between 2 groups (dominant or recessive model) for each polymorphism with adjustment for age, gender, BMI, smoking status, and the prevalence of hypertension, diabetes mellitus and dyslipidemia. Data for serum creatinine and eGFR are the means ± SD. P-values of <0.05 are shown in bold.

a Values in parentheses are numbers of measurements. *ALPK1*, α-kinase 1 gene; *BTN2A1*, butyrophilin, subfamily 2, member A1 gene; eGFR, estimated glomerular filtration rate; BMI, body mass index; SNP, single nucleotide polymorphism.
